# A Blast From the Past: Abdominal Wall Amyloidosis Due to Enfuvirtide Injections

**DOI:** 10.7759/cureus.43126

**Published:** 2023-08-08

**Authors:** Cecilia Costiniuk, Anne-Marie Bourgault, Chelsea Maedler-Kron, Liane Feldman

**Affiliations:** 1 Medicine, Research Institute of the McGill University Health Centre, Montreal, CAN; 2 Infectious Diseases/Medical Microbiology, Royal Victoria Hospital/McGill University Health Centre, Montreal, CAN; 3 Laboratory Medicine/Pathology, Royal Victoria Hospital/McGill University Health Centre, Montreal, CAN; 4 Surgery/General Surgery, Montreal General Hospital/McGill University Health Centre, Montreal, ASM

**Keywords:** amyloidosis, skin nodules, hiv, injection site reactions, enfuvirtide

## Abstract

Enfuvirtide was the first marketed fusion inhibitor that worked by binding to glycoprotein 41 of the HIV envelope, preventing the conformational changes required for HIV fusion with CD4+ T cells. Due to its novel mechanisms of action, it was frequently used in the past as part of regimens for the indication of multi-class-resistant HIV until newer oral agents emerged. Here, we describe the case of a 40-year-old man who used enfuvirtide injections from 2012 to 2017 inclusive for multi-class-resistant HIV until he presented in 2021 with an abscess overlying a right lower quadrant mass requiring drainage via pigtail. Congo red stain of the tissue showed positive apple green birefringence on amorphous material after polarization, enabling the diagnosis of enfuvirtide-induced amyloidosis. The patient experienced significant improvement following surgical excision of the cysts and nodules. This case demonstrates that sequelae of injection site reactions can persist for many years following the cessation of enfuvirtide injections.

## Introduction

Enfuvirtide, a 36-amino-acid peptide, was the first marketed fusion inhibitor that worked by binding to glycoprotein 41 of the HIV envelope, preventing the conformational changes required for HIV fusion with CD4+ T cells [1]. Due to its novel mechanisms of action, it used to be a part of regimens for multi-class-resistant HIV until newer oral agents emerged. Various injection site reactions (ISRs) were common with enfuvirtide use [2]. In fact, ISRs were the most common limiting factor to enfuvirtide injections, with 98% of individuals reporting induration, 89% experiencing erythema, and 76% experiencing nodules and cysts [3]. However, only 3% of individuals stopped the medication due to severe ISRs. Although rare, the specific incidence of cutaneous amyloidosis among ISRs due to enfuvirtide is unknown. Cutaneous amyloidosis results from the deposition of insoluble proteins or peptides with a characteristic beta-diffraction pattern extracellularly [4], and has been associated with enfuvirtide use. Here, we present the case of a 40-year-old man who used enfuvirtide injections from 2012 to 2017 for the indication of multi-class-resistant HIV until he presented in 2021 with an abscess overlying an abdominal wall skin mass. Pathology enabled the diagnosis of enfuvirtide-induced amyloidosis.

## Case presentation

A 40-year-old indigenous man, with HIV since the age of 16, presented to the outpatient HIV clinic in 2021 with a two-week history of increasing pain and redness overlying a right lower quadrant (RLQ) abdominal mass. Other past medical history included obesity, hypertension, gastroesophageal reflux disease, and polysubstance use. Regarding the latter, he smoked tobacco, consumed cannabis heavily, and used cocaine. Although he endorsed previous history of intravenous drug use, he denied ever injecting materials into his abdominal wall. Due to poor medication adherence and multi-class-resistant HIV, he was on a regimen of etravirine, tenofovir, emtricitabine, ritonavir, darunavir, and dolutegravir since 2017. Other medications included pantoprazole and amlodipine. Before 2012, his antiretroviral regimens included atazanavir, lamivudine, and ritonavir-boosted indinavir (December 2000); tenofovir, emtricitabine, and ritonavir-boosted lopinavir (July 2008); and tenofovir disoproxil, emtricitabine, ritonavir-boosted darunavir, and etravirine (September 2010). Enfurvitide was added to his regimen in 2012.

He was off treatment since March 2017. In June 2017, we was started on tenofovir, emtricitabine, ritonavir-boosted darunavir, etravirine, and dolutegravir. During this time, he self-discontinued medications on several occasions, resulting in multiple treatment interruptions.

From 2012 to 2017 inclusive, he received enfuvirtide 90 mg twice daily injected subcutaneously, resulting in chronic abdominal wall nodules. In 2017, he developed an abscess overlying the RLQ mass requiring drainage via pigtail. The culture showed 1+ *Aspergillus niger*, which was deemed a contaminant. Although bacterial cultures were negative, a bacterial superinfection was still deemed probable. Therefore, he received intravenous cefazolin followed by oral cefadroxil treatment for a total of 14 days. This duration of treatment was suggested to eliminate any residual infection following drainage.

**Figure 1:**
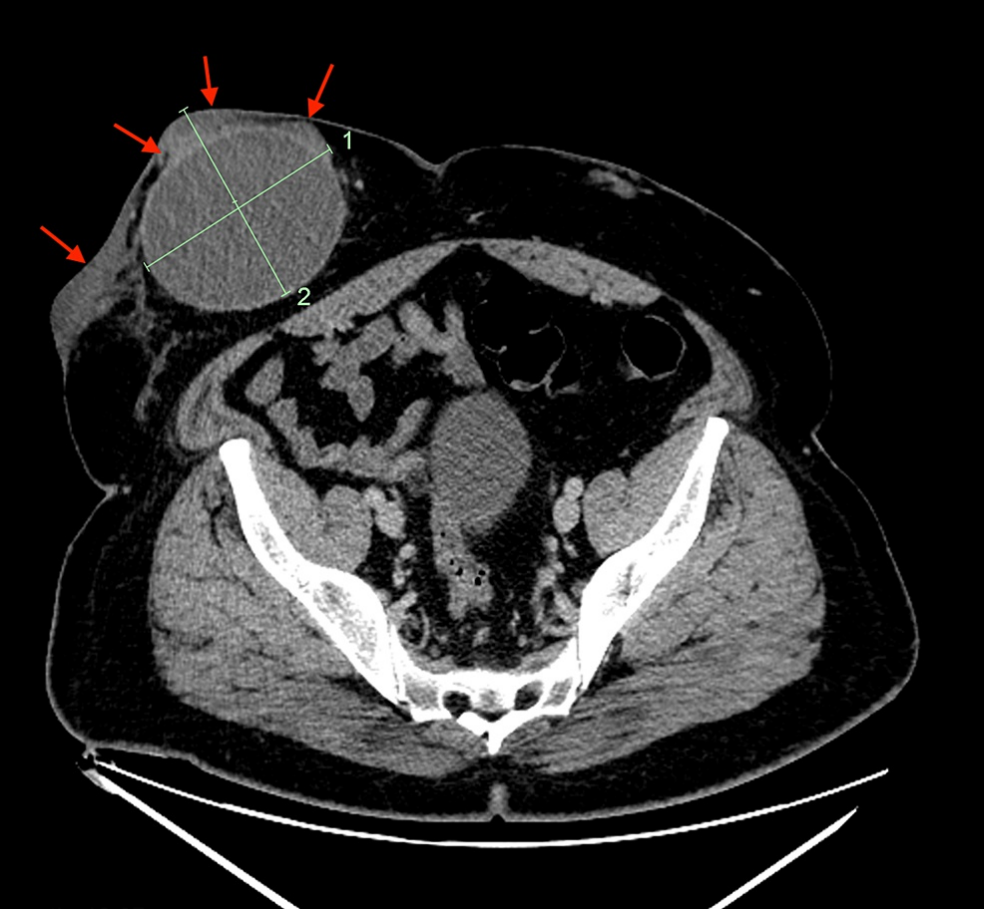
Computed tomography scan showing multiple large abdominal wall nodules with a right lower quadrant cystic mass. Computed tomography scan showing multiple large abdominal wall nodules with a right lower quadrant cystic mass measuring 10.5 × 8.8 × 10.7 cm, stable since 2017. Superficial to this cyst was skin and subcutaneous tissue thickening with fat stranding (red arrows) and a round density within the right lower quadrant cyst.

At this 2021 encounter, he was afebrile and his vital signs were stable. The skin overlying the RLQ mass was firm and warm. He had smaller, soft lower quadrant (LLQ) nodules. White blood cell count was normal, and C-reactive protein was 20.10 mg/L. The most recent viral load a year earlier was undetectable, the CD4 count was 602 cells/mm^3^ (22%), and the CD4/CD8 ratio was 0.5. A CT scan showed multiple large abdominal wall nodules with an RLQ cystic mass measuring 10.5 × 8.8 × 10.7 cm, which was stable since 2017. Superficial to this cyst was skin and subcutaneous tissue thickening with fat stranding and a round density within the RLQ cyst (Figure 1).

He was started on piperacillin-tazobactam, underwent incision and drainage, and then discharged with oral cefadroxil for two weeks. Once again, despite negative bacterial cultures, the clinical presentation appeared consistent with bacterial superinfection. The duration of therapy was suggested to treat any residual infection following drainage.

**Figure 2:**
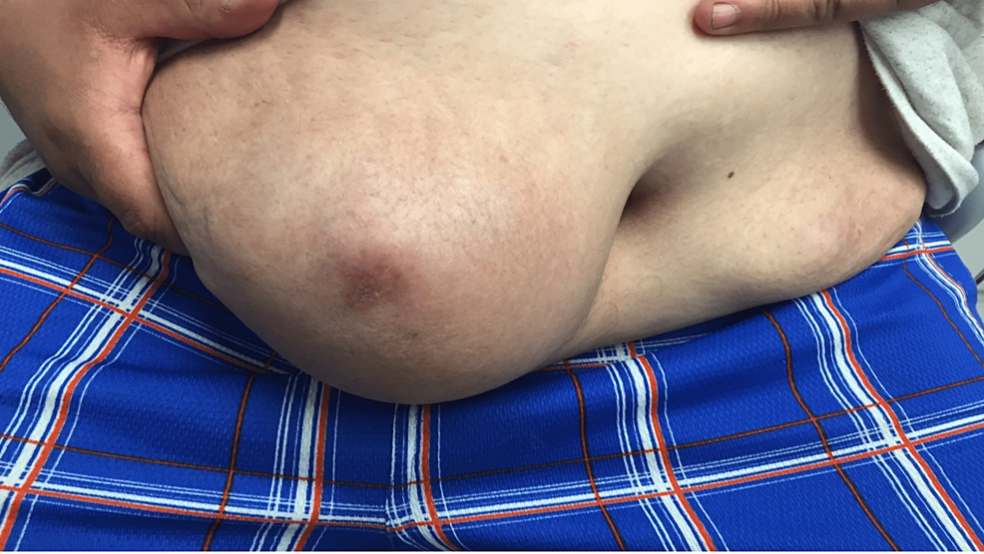
Large, right lower quadrant mass which had been present since 2017. The mass was generally painless but would become superinfected. It was very bothersome to the patient due to aesthetic reasons and was tender when accidentally knocked against objects.

He returned in 2022 complaining that the RLQ mass was affecting his quality of life. He had an RLQ 15 cm subcutaneous erythematous mass which was tender, warm, and fluctuant at the periphery (Figure 2).

Intraoperatively, he had multiple, encapsulated anterior wall cysts, the largest 12 cm in the RLQ, and two smaller LLQ cysts which were all removed. The RLQ capsule was inadvertently punctured and released old blood and thickened debris, which were drained. The LLQ cysts were also dissected and removed. Nodular tissue culture, including bacterial, mycobacterial, and fungal cultures, returned negative. Polymerase chain reaction (PCR) for 16sRNA revealed *Corynebacterium glucuronolyticum* in broth only, thought to be a contaminant. PCR for mycobacteria and fungi was also negative.

**Figure 3:**
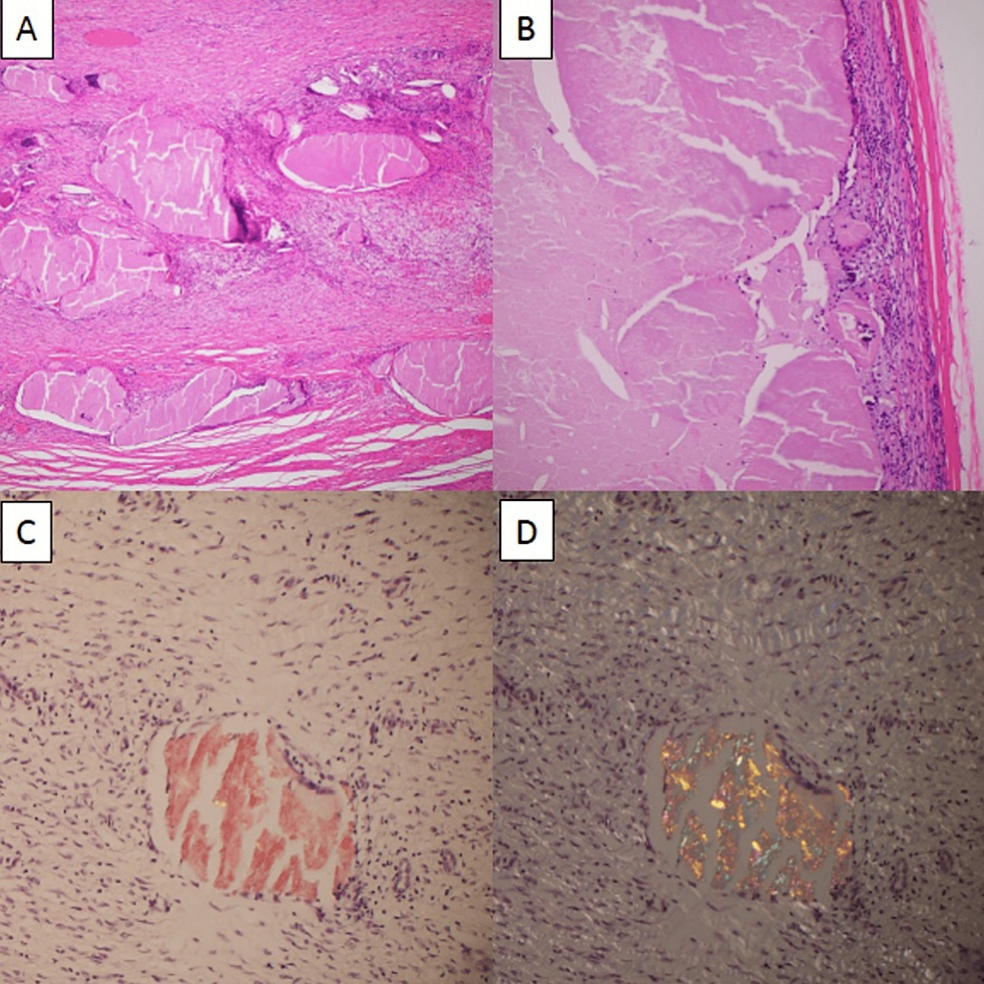
Pathology of the right lower quadrant mass. (A) Low power (2×, hematoxylin and eosin) showing subcutaneous deposits amorphous of eosinophilic amyloid material. (B) High power (10×, hematoxylin and eosin) of cystic encapsulated cavity with xanthogranulomatous response to iatrogenic amyloid deposits. (C, D) High power (20×, Congo red) of amyloid deposits polarized to show apple green birefringence.

On gross exam, the RLQ wall nodule was firm, encapsulated, and measured 2.5 × 1.7 × 1.5 cm, with a smooth outer surface. The RLQ cyst was 7.0 × 6.0 × 6 cm in size, with a smooth 0.5 cm thick wall. The rough inner surface had an attached necrotic mass. The cyst contained amorphous proteinaceous debris. On pathology, the tissue was negative for malignancy and opportunistic microorganisms, with Periodic acid-Schiff, Grocott methenamine silver, Ziehl-Neelsen, and Gram stains all negative. The adjacent soft tissue had a xanthogranulomatous response and chronic fibrosis and inflammation. Congo red stain showed positive apple green birefringence on amorphous material after polarization (Figure 3).

Pathology suggestive of amyloidosis believed to be due to enfuvirtide injections enabled the diagnosis to be made. Following the surgical excision, our patient has not attended any follow-up appointments.

## Discussion

Amyloidosis is a heterogeneous disease associated with the deposition of insoluble proteins or peptides with a characteristic beta-diffraction pattern extracellularly [4]. Nearly 30 different extracellular fibril proteins cause diseases in humans [4]. Case reports involving enfuvirtide-induced amyloidosis have previously been published [5-7], and one study demonstrated that the amyloid structure is composed of the enfuvirtide peptide itself [8]. While extensive deposition of the drug demonstrates that it is causative in amyloidogenesis, the precise pathogenesis involving the conversion of enfuvirtide peptide into fibrillar aggregates of amyloid has not been elucidated [8]. As precursor protein persists within amyloid masses, the origin can sometimes be confirmed immunohistochemically [8].

Wallace et al. conducted a clinical and histopathological study to analyze in detail the histopathological changes associated with enfuvirtide ISR in four patients [9]. Biopsies obtained at various time points post-injection of epidermal, derma3l, and subcutaneous connective tissue revealed significant changes in dermal connective tissue, with alteration in collagen being the most striking feature and resembling a morphea/scleroderma-like process [9]. The changes persisted beyond the cessation of enfuvirtide over a year [9].

To our knowledge, insulin and enfuvirtide are the only two drugs that have been reported to cause iatrogenic amyloidosis [10]. Specific anti-insulin antibodies may be used in histochemical studies by reacting against the insulin amino acid sequence which remains unaltered during pegylation [2]. In insulin ISR, a histopathologic examination demonstrates eosinophilic and amorphous masses in the deep dermis which stain positive for Congo red, amyloid P substance, and anti-human insulin antibody [11]. In vivo, amyloid deposits have been detected in the skin and bronchoalveolar regions because of injected insulin and inhaled insulin, respectively [12]. It is thought that the concentration of insulin achieved at a specific site is the most important risk factor for its development. Amyloidosis has been associated with inconsistent insulin release [2]. It is unknown if amyloidosis or skin reaction are associated with poorer absorption of enfuvirtide and poorer HIV control. Our patient’s viral loads between 2012 and 2017 were poorly controlled. It is unclear if poor viral control was due to poor bioavailability versus poor medication adherence, as the patient’s presence at medical appointments was limited. Although the number of cases reported in the literature is relatively few, surgical resection appears to be the optimal management strategy. To our knowledge, no other cases of enfuvirtide ISR have presented with multiple nodule cysts with secondary infection and abscess formation have been reported. Similarly, to our knowledge, surgical resection appears curative and does not lead to chronic abscess formation. As our patient has not attended any follow-up appointments following the surgical excision, we cannot confirm if he has experienced any recurrences despite surgical treatment.

## Conclusions

This case illustrates the fact that ISRs from enfuvirtide can persist for many years despite stopping the injections. It also illustrates that lesions can become very large and cysts may become superinfected. Ruling out malignancies such as Kaposi’s sarcoma and mycobacterial infections is critical. Surgical excision is the definitive treatment.
